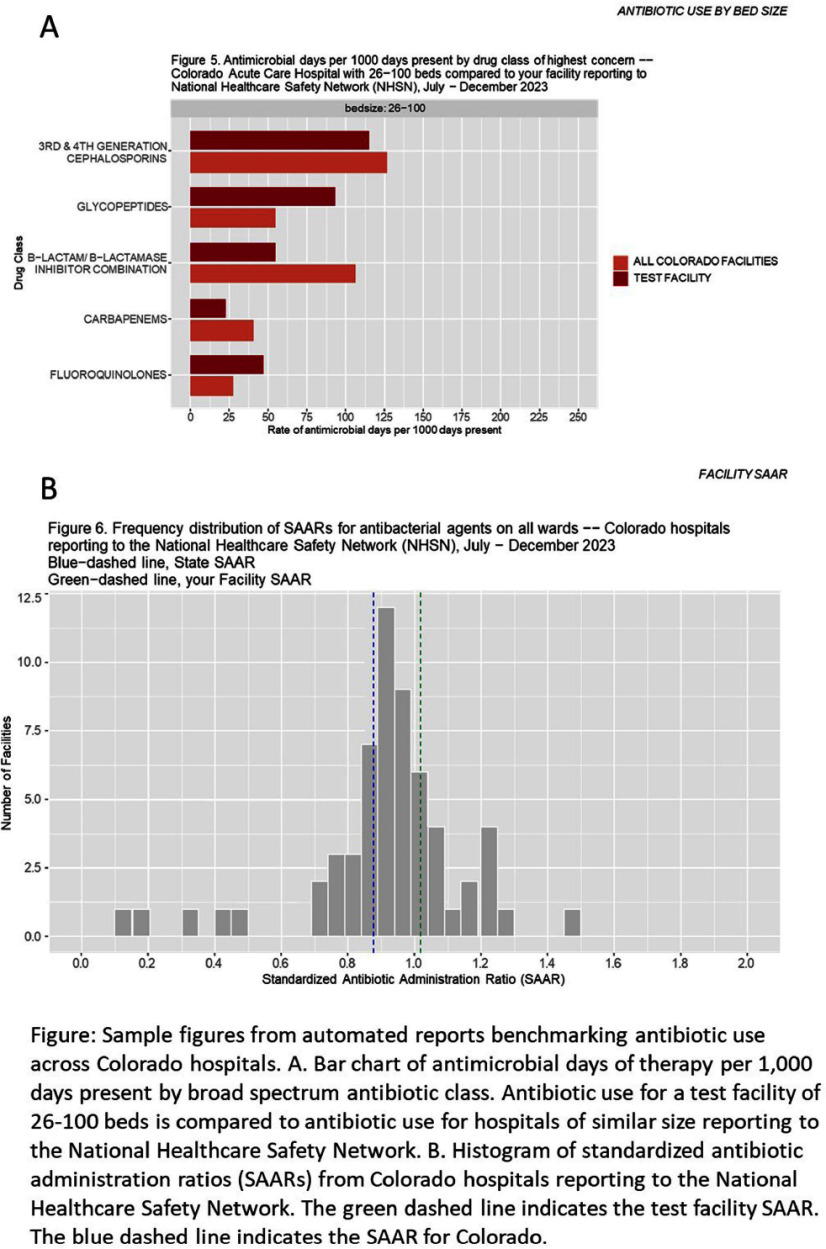# Public health delivery of automated antibiotic use and adherence reports to hospital antibiotic stewardship programs in Colorado

**DOI:** 10.1017/ash.2025.384

**Published:** 2025-09-24

**Authors:** Rachel Schaefer, Christopher Czaja, Lauren Biehle, Michael Bozzella, Joana Dimo, Leigh Anne Bakel, Sarah Parker, Matthew Weber

**Affiliations:** 1 Colorado Department of Public Health and Environment; 2 Colorado Department of Public Health and Environment; 3 Colorado Department of Public Health and Environment; 4 University of Colorado School of Medicine; 5 Children’s Hospital Colorado; 6 Children’s Hospital Colorado/UCSOM; 7 University of Colorado School of Medicine

## Abstract

**Background:** Tracking and reporting antibiotic use (AU) are core elements of hospital antibiotic stewardship. However, not all hospitals have the capacity to analyze AU data. The Colorado Department of Public Health and Environment and Children’s Hospital Colorado generated two automated reports of hospital AU to support antibiotic stewardship programs (ASPs) across Colorado. **Methods:** The first report was an AU feedback report that summarized data from the National Healthcare Safety Network (NHSN) Antimicrobial Use and Resistance Module. Distributed twice per year, it included hospital rates of AU for high-volume and broad-spectrum antibiotics compared to rates from facilities of similar type, bed size, and geographic region (Figure). We solicited feedback from users of the AU feedback report via REDCap survey. The second report (pathway adherence) summarized adherence to treatment guidelines for adult and pediatric community acquired pneumonia (CAP), adult urinary tract infections (UTI), and adult skin/ soft tissue infections (SSTI). The pathway adherence report used self-reported, deidentified, case-level data entered by hospitals into a REDCap survey, and incorporated individualized review and expert guidance to assist ASP interventions. We analyzed all data and created custom PDFs in R-Studio and R-Markdown. **Results:** Between May 2023 and November 2024 we distributed 272 AU feedback reports to 52/55 (94%) acute care hospitals (ACH) and 23/33 (69%) critical access hospitals (CAH) in Colorado. Participating hospitals were distributed across the state and had a median (range) bed size of 49 (8– 828). Among 14 hospitals that provided feedback, most users said AU feedback reports included meaningful visual comparisons and helpful data quality checks. Many facilities responded that they shared the AU feedback reports with hospital leadership, pharmacists, prescribers, infection preventionists, nurses and laboratory personnel, in addition to ASPs and steering committees. In October 2024, we distributed 34 AU adherence reports to seven ACH and six CAH, including: four pediatric CAP, ten adult CAP, twelve UTI, and eight SSTI reports. **Conclusion:** Automated AU feedback and adherence reports were feasible, scalable, and well-received. They fostered an opportunity for public health to connect with hospital ASPs and provide 1:1 mentorship. Centrally-developed, individualized reports provide an analytic service to equip ASPs with concise, comprehensive summaries of their hospital’s AU.

**Figure:**